# Developing a quality and safety assessment framework for Iran’s military hospitals

**DOI:** 10.1186/s12913-024-11248-w

**Published:** 2024-07-02

**Authors:** Nader Markazi-Moghaddam, Mojgan Mohammadimehr, Mahdi Nikoomanesh, Ramin Rezapour, Sanaz Zargar Balaye Jame

**Affiliations:** 1 Critical Care Quality Improvement Research Center, Shahid Modarres Hospital, Shahid Beheshti University of Medical Sciences, Tehran, Iran; 2 Department of Health Management and Economics, School of Medicine, AJA University of Medical Sciences, Tehran, Iran; 3 Infectious Diseases Research Center, Aja University of Medical Sciences, Tehran, Iran; 4 Health Management and Economics Research Center, Iran University of Medical Sciences, Tehran, Iran; 5 Tabriz Health Services Management Research Center, Tabriz University of Medical Sciences, Tabriz, Iran

**Keywords:** Assessment Framework, Quality, Safety, Military Hospital, Iran

## Abstract

**Background:**

The first crucial step towards military hospitals performance improvement is to develop a local and scientific tool to assess quality and safety based on the context and aims of military hospitals. This study introduces a Quality and Safety Assessment Framework (Q&SAF) for Iran’s military hospitals.

**Methods:**

This is a literature review which continued with a qualitative study. The Q&SAF for Iran’s military hospitals was developed initially, through a review of the WHO’s framework for hospital performance, literature review (other related framework), review of military hospital-related local documents, consultations with a national and sub-national expert. Finally, the Delphi technique used to finalize the framework.

**Results:**

Based on the literature review results; 13 hospital Q&SAF were identified. After reviewing literature review results and expert opinions; Iran’s military hospitals Q&SAF was developed with 58 indictors in five dimensions including clinical effectiveness, safety, efficiency, patient-centeredness, and Responsive Management (Command and Control). The efficiency dimension had the highest number of indictors (19 indictors), whereas the patient-centered dimension had the lowest number of indices (4 indictors).

**Conclusion:**

Regarding the comprehensiveness of the developed assessment framework due to its focus on the majority of quality dimensions and important components of the hospital’s performance, it can be used as a useful tool for assessing and continuously improving the quality of hospitals, particularly military hospitals.

## Background

Military healthcare structures, particularly military hospitals, play an important role in achieving the health system’s goals and responding to the population health needs by supporting and providing medical services to the armed forces in military operations as well as assisting the civilian healthcare system [1].

In the hospital, due to the importance of services and dealing with human lives, quality assurance and improvement have become increasingly crucial [2]. Quality is a broad and multifaceted concept including technical competence, access to services, effectiveness, interpersonal relationships, efficiency, continuity and safety [3]. Quality improvement has gained increased attention in recent decades as an approach to increase service effectiveness, particularly in developing countries, and significant efforts have been made to improve the quality of healthcare services [4]. Service quality assessment is the first step to quality improvement [5]. Quality Assessment Framework (QAF) (including quality dimensions and assessment indicators) is one of the standard quality assessment methods [6]. QAFs are developed in accordance with health system requirements, strategies, and objectives. Each country has proposed different dimensions and indicators for quality assessment [7–9]. The USA has proposed the dimensions of efficiency, access, health system infrastructure, patient-centeredness, effectiveness, safety, coordination, and timeliness to assess quality of health care [10]. The World Health Organization (WHO) Regional Office for Europe has introduced Performance Assessment Tools for Hospital (PATH) with six dimensions including clinical effectiveness, staff orientation, responsible governance, safety and patient-centered [11]. The variation in QAFs demonstrates the necessity of considering each health system needs, strategies, goals, and service delivery infrastructure when developing these frameworks [12, 13].

To measure the quality and safety of hospitals and create the basis for analyzing the strengths and weaknesses regarding hospital performance, it is crucial to acquire a local and scientific tool based on the hospital conditions [9]. Military hospitals should be assessed based on their unique indicators due to their unique missions and services related to receipting special patients or dealing with biological, chemical, and nuclear disasters [14]. It is necessary to pay special attention to the organizational structure, manpower, type and amount of equipment in developing the performance assessment of military hospitals [15, 16].

To the best of our knowledge, there is currently no local and national framework for assessing the Iran’s military hospitals, while majority of countries in the world use a specific national framework to assess the performance and quality of hospital. This research seeks to develop a comprehensive and scientific framework for measuring multiple dimensions of quality using worldwide experiences. Hospital managers can acquire a comprehensive insight of current performance with the assistance of the data provided by this framework. This study was conducted to develop a Quality and Safety Assessment Framework (Q&SAF) for Iran’s military hospitals through an adjusted framework from WHO.

## Methods

**Fig. 1:**
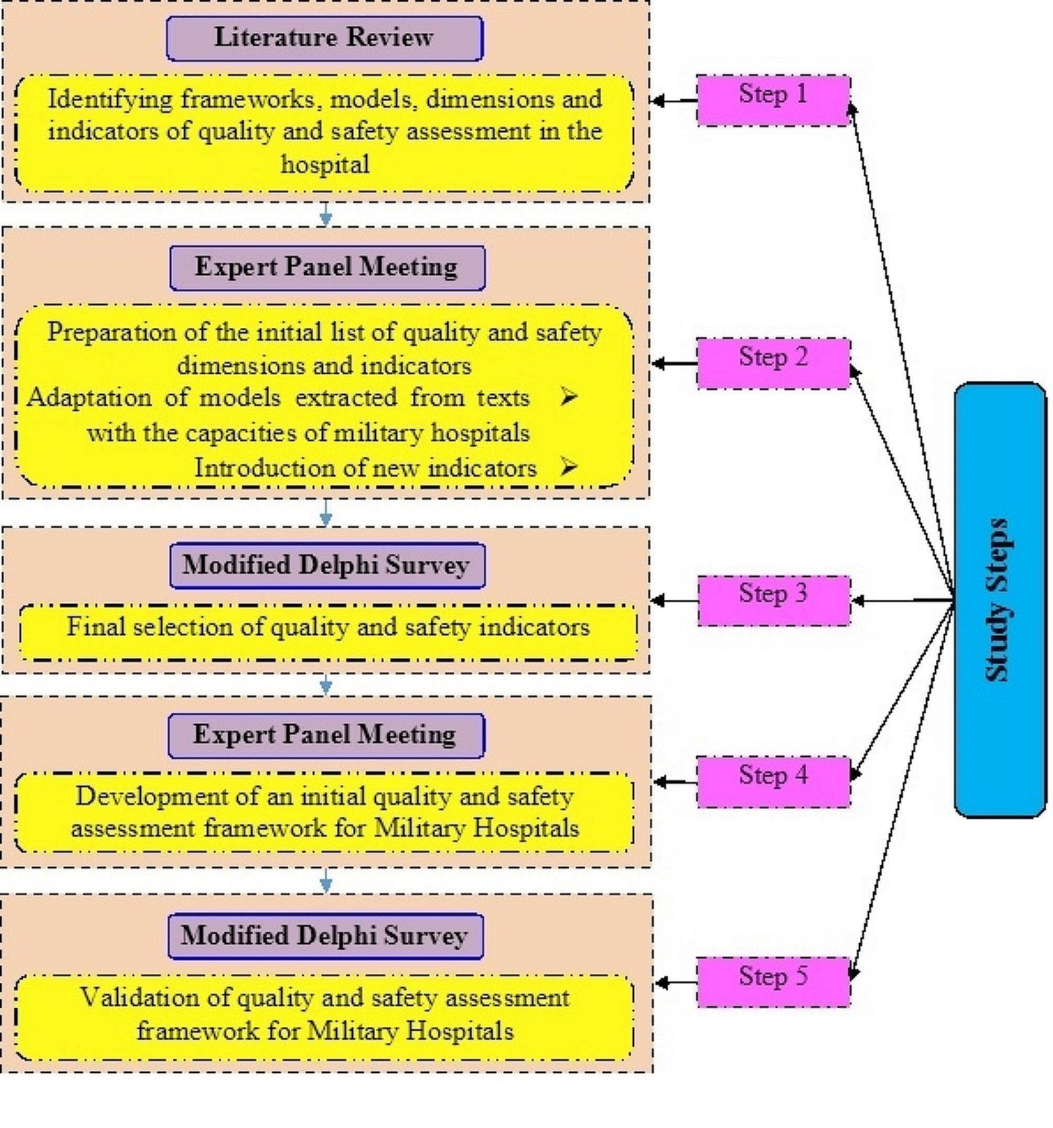
Irans military hospitals quality and safety assessment framework development flow

This is a qualitative study which was conducted in 2023. In order to develop a Q&SAF for Iran’s military hospitals, first, the quality dimensions and indicators as well as the frameworks and models in the scientific literature were identified (Literature review). Then, the expert panels held meetings to adapt the models and frameworks to the local conditions of the country and military hospitals as well as to introduce new indicators in accordance with the potentials and capacities of military hospitals (Expert panel). The results of the expert panel meetings led to the preparation of the initial list of quality and safety assessment dimensions and indicators. After preparing the initial list of indicators, in order to select the final indicators and reach a consensus regarding the final indicators, a qualitative survey was used (Modified Delphi survey). In the next step, the indicators selected based on the expert’s opinion were categorized quality dimensions, and the initial Q&SAF for Iran’s military hospitals was developed (Expert panel). In the last step; content validity index and Modified Kappa were used to finalize and validate the developed framework (Modified Delphi survey). The steps of developing the framework are indicated in Fig. 1.

### Step 1: Identifying frameworks, models, dimensions and indicators of quality and safety assessment in the hospital

The methodology of overview was used in order to identify the models and frameworks for assessing the quality and safety in the hospital, as well as the indicators associated with each framework. Databases of PubMed, Scopus, web of science, and websites related to the WHO using related keywords and their Persian equivalents in Persian databases in the period from 2000 to 2023 were reviewed. The keywords included quality indicator, quality assessment, quality evaluation, quality assurance, performance indicator, standard, quality improvement, Hospital, health center, health facility, inpatient car, model, framework, project, plan. Additionally, a manual search of specialized journals and references of selected articles, organizational reports and other available information sources was done.

The studies that were developed for the hospital environment and also provided a comprehensive framework for assessing quality and safety (considering all aspects of quality and safety and not focusing on a specific dimension or service) were selected for review. Due to the variety of studies, papers written in languages other than Persian and English, studies conducted in settings outside of hospitals, and studies which focused on the quality of specific service or procedure were excluded from the review. Review and screening of studies was done according to Prisma guideline [17] and using Endnote software. In this step, the functional dimensions, the list of indicators and the scope of the identified frameworks were extracted.

### Step 2: Preparation of the initial list of quality and safety dimensions and indicators

In this step, the frameworks and models extracted from the literature were reviewed according to the capacities and potentials of military hospitals as well as the condition of Iran’s health system. A qualitative study (expert panel) was used for this objective. Following an initial meeting with experts, the dimensions of the Q&SAF for Iran’s military hospitals were selected. These dimensions were those that were most frequent among the identified frameworks and were most consistent with the conditions of Iranian hospitals. Next, the assessment indicators related to each of the dimension were reviewed. The primary criteria for selecting indicators included: the ability to measure the indicator in the hospital, the importance of the indicator, and the relevance of the indicator to the operational processes of the military hospitals.

Members of the expert panel included individuals with an experience in hospital performance assessment and the quality and safety improvement, as well as other individuals and academic members with related knowledge. These members were selected through the heterogeneous purposeful sampling technique (participants with maximum diversity).

Reviewing dimensions and indicators was done during two face-to-face meetings (Skype platform) for about 1.5 h. During these meetings, in addition to reviewing and selecting the dimensions and indicators extracted from the literature, new indicators suitable to the conditions of military hospitals were also introduced by the experts. In this way, a list of quality and safety assessment indicators was prepared.

### Step 3: Final selection of quality and safety indicators

After preparing the initial list of indicators based on the results of the previous steps, a modified Delphi survey [18, 19] was used to reach a consensus about the indicators.

A purposeful sampling technique (according to the type of dimensions and indicators) was used to select participants of survey. The inclusion criteria for the participants included officials and managers of military hospitals and vice chancellor of treatment with at least 5 years of experience, policy makers of the Ministry of Health, and academic members in the fields of health and services management and health economics, health emergencies disaster and health information management.

The selection criteria of the indicators according to the criteria introduced by the WHO [20] included: the importance, feasibility and relevance of the indicator. Each of indicator scored between 1 and 5 based on the three criteria. The indicators were selected using the following parameters: indications with an average of less than 2 were disqualified, those with scores between 2 and 3.5 were returned to the second round of Delphi, and those with a score of 3.5 or more were accepted as the final indicators.

### Step 4: Development of an initial Q&SAF for Military Hospitals

The initial framework was developed by the research team and experts based on the findings of the literature review and the qualitative part of the study. To develop the initial framework; the selected final indicators were classified in the selected dimensions in the second step. Also, in this step, for each dimension, related sub-dimensions were defined. The selection process for member of expert panel was similar to the second step.

### Step 5: Validation of Q&SAF for military hospitals

The validity of the developed framework was assessed based on the opinions of experts. Accordingly, the initial framework with a detailed description of dimension and indicators sent to 10 experts throughout the Delphi questionnaire. To assess the validity of the framework, 10 items were evaluated. These items included (1) Applicability of the framework (2) Adaptation of the developed framework to the upstream documents (3) Ability to accept the framework by stakeholders (4) Efficiency (5) Flexibility (6) Effectiveness (7) Simplicity (8) Coherence and integration between framework dimensions (9) Comprehensiveness and (10) Overall.

In order to confirm the validity of the framework, modified content validity index and modified Kappa were used. This method was presented by Polit et al. in 2007 [21]. The following formulas were used to calculate Kappa.

$${p_c} = \left[ {\frac{{N!}}{{A!(N - A)!}}} \right]{.5^N} \Rightarrow k* = \frac{{I-CVI - {p_c}}}{{1 - {p_c}}}$$

N = Number of Experts.

A = the number of experts with score of a completely agree and agree.

Experts scored each of the items based on a 4-point Likert scale (completely agree to completely disagree). According to Polit et al.‘s proposal, Kappa lower than 0.40 be considered (necessary), between 0.6 and 0.74 (good) and above0.74 (Excellent).

## Results

**Fig. 2:**
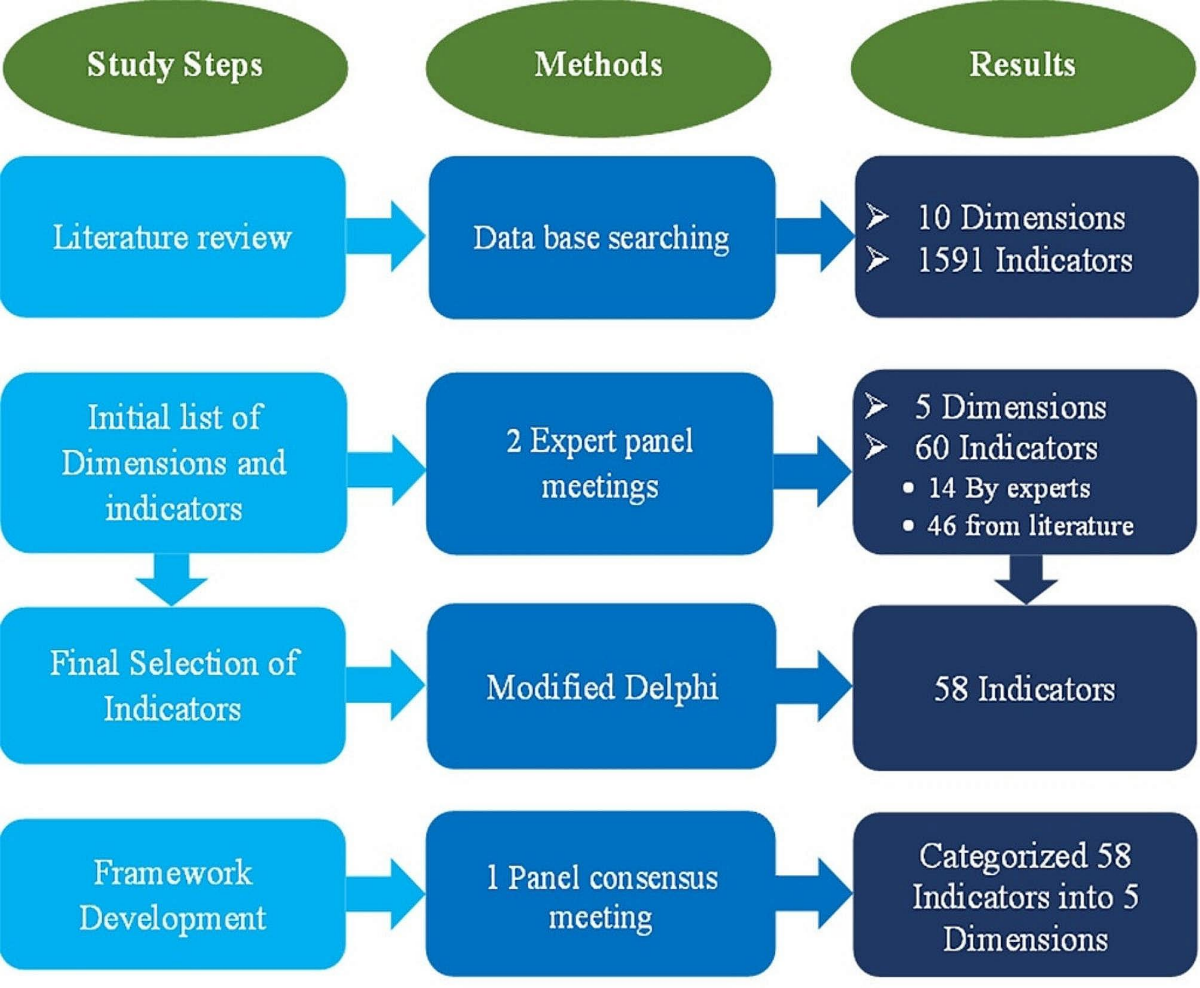
The results of the development steps of Irans Military Hospitals Quality and Safety Assessment Framework

The Q&SAF for Iran’s military hospitals was developed in five main steps. During the first step, 13 frameworks, 10 dimensions, and 1591 indicators related to each framework were extracted. In the next step, 5 dimensions and 60 indicators were selected based on the findings of the literature review and the recommendations of experts. Based on the results of the Delphi survey, 2 indicators were removed from the 60 indicators and finally 58 indicators were selected. The selected indicators were categorized in the five dimensions (Fig. 2). In the last step, ten experts were asked to assess validity of the framework, and after receiving their feedback, the estimated Kappa index for the framework was 8.9 out of 10.

### Step 1: Identifying frameworks, models, dimensions and indicators of quality and safety assessment in the hospitals

After screening the studies and reports extracted from the literature, finally; 13 frameworks along with 10 dimensions and 1591 indicators were identified. The dimensions were compared in order to determine their frequency (Table 1). The identified indicators were initially screened and after removing duplicate and unrelated indicators and merging similar ones, finally 137 indicators were selected.

**Table 1:** Comparison of quality and safety dimensions in hospitals

Dimensions	WHO-PATH [22]	ACHS [23]	BQS Germany [24]	NHS [25, 26]	COMPAQH [27]	CHRP [28]	JCAHO [29]	OHA [30]	HMIS [31]	QIP [32]	NIP [33]	Netherland [34]	Switzerland [35]
Clinical Governance	*	*	*	*	*	*	*			*	*	*	*
Staff Orientation	*				*	*							
Efficiency	*	*				*	*	*		*	*	*	*
Responsive governance	*							*	*				*
Safety	*	*				*	*			*	*	*	*
Patient Centeredness	*				*		*	*		*	*	*	*
Appropriateness						*							
Access						*							
Quality									*				
Timeliness		*							*				

### Step 2: Preparation of the initial list of quality and safety dimensions and indicators

Experts’ meetings with the participation of 9 experts (3 experts from the army hospital assessment and monitoring department, 6 academic faculty members (2 health management specialist with a focus on service quality assessment, 2 health information management specialist and 2 health emergencies disaster specialist) were held. In addition to the results of the literature review, the list of performance assessment indicators of military hospitals and other related documents about Iranian hospital performance assessment, were also reviewed by an experts’ panel. Based on the results of expert panel meetings, 5 dimensions including clinical effectiveness, safety, efficiency, patient-centeredness and Responsive Management (Command and Control) along with 60 quality and safety assessment indicators (14 indicators by experts and 46 indicators from literature) according to conditions and potential of Iran’s military hospitals were selected. Among the dimensions, the Responsive Management (Command and Control) dimension specifically focuses on the processes and performance of military hospitals.

### Step 3: Final selection of quality and safety indicators

The initial list of indicators was reviewed by experts through the modified Delphi survey. The participants in the Delphi survey included 2 experts from the regional office of the WHO, 2 faculty members of the Army University of Medical Sciences, 4 faculty members of medical sciences universities across the country, and 2 hospital managers. Based on the results of the Delphi survey; finally, 58 indicators (out of 60 indicators) scored higher than 3.5 and were selected as final indicators (Table 2).

### Step 4: Development of an initial Q&SAF for military hospitals

Expert panel meetings were held to review the final indicators and classify them among the dimensions. Also, in these meetings, sub-dimensions were defined for each dimension. Finally; The Q&SAF for military hospitals was developed with 5 dimensions and 15 sub-dimensions (Table 3). Among the dimensions, the most indicators were related to the efficiency dimension (19 indicators) and the lowest indicators were related to the patient-centered dimension (4 indicators).

Also, among the following sub-dimensions; the most indicators are related to the sub-dimension of financial performance (9 indicators) and the lowest indicators are related to the information security and management (1 indicator), environmental safety management (1 indicator) and combat medicine and military health management (1 indicator).

### Validation of Q&SAF for military hospitals

The developed framework was sent to 10 experts (similar to the step 3) in order to validate it. Due to the obtained score above 0.74 in all 12 criteria of the questionnaire, the Delphi survey was completed in the first round and the Q&SAF for Military Hospitals was finalized (Table 4).

**Table 2:** List of quality and safety assessment indicators in Iran’s military hospitals

No.	Indicators Name	Average Score	No.	Indicators Name	Average Score
1	Percentage of repeat surgical procedures	4.7	16	Postoperative respiratory failure	4.67
2	Appropriateness of prophylactic antibiotic use	4.7	17	Postoperative sepsis	4.7
3	Discharge against medical advice*	5	18	Wrong surgery rate (wrong side, wrong body part, or wrong person)	4.67
4	Surgery postponed or canceled	4.97	19	Rate of Pressure Ulcers/bed sores*	5
5	Percentage of assigned patients in the emergency department within six hours	4.67	20	Staff burnout*	4.67
6	The time interval between the patient entering the emergency ward and the start of treatment (stroke and heart attack)	4.63	21	Hand hygiene compliance rate	5
7	Unplanned readmissions	4.7	22	Number of work-related injuries*	4.63
8	Rate of return to ICU	5	23	Employee sick leave rates*	4.7
9	Mortality rate in intensive care unit	5	24	Needle stick events	5
10	Perioperative mortality/ Number of deaths after surgery	5	25	Percentage of HW immunized for Hepatitis B (completed the 3 doses) *	5
11	The pure rate of hospital mortality	5	26	Number of OR cases cancelled	4.7
12	Hospital-acquired infections	4.7	27	Ambulatory surgery rate (medical acute care)	4.1
13	Prevalence of sentinel events (28 common events)	4.7	28	Ratio of physicians to bed	5
14	Medical errors per sector (detected by GTT)	4.63	29	Ratio of nurses to bed	5
15	The number of falling patients	4.63	30	Average length of stay	5
31	Percent of patients admitted on day of surgery	4	45	% of Customers/Patients Complaints by service types	5
32	Bed occupancy rate	4.97	46	Average handling time for patients’ complaints by service types*	5
33	Bed turnover interval	5	47	Patient satisfaction	5
34	Average inventory in stock, for pharmaceuticals, blood products, surgical disposable equipment*	4	48	Patient Experience	5
35	Operating room unused sessions / Operating Room utilization rate	4.67	49	Staff absenteeism (more than 42 days)	5
36	The ratio of net income (revenues/expenses) to total revenues	5	50	Staff turnover rate	5
37	Personnel expenditure as % of total expenditure	4.7	51	Jab satisfaction rate	4.7
38	Medicine expenditure as % of total expenditure	4.7	52	Number training hours on total number of working hours	5
39	Administrative service expenditure as % of total expenditure	4.37	53	Training budget on total budget dedicated to staff	4.67
40	Equipment maintenance expenditure as % of total expenditure	5	54	The improvement rate of the accreditation score in the accident and disaster management sector*	4.67
41	Consumption expenditure as % of total expenditure	4.67	55	The average improvement rate of the three indicators of hospital safety*	4.67
42	The average cost of a patient’s hospitalization day	5	56	The improvement rate of the accreditation score in the health information technology and management sector*	4.67
43	insurance deductible percentage*	5	57	The improvement rate of the accreditation score in the environmental health sector*	4.67
44	Cost of outpatient services per patient	4.33	58	The improvement rate of the accreditation score in the combat medicine sector*	5

* Indicators suggested by experts

**Table 3:** Quality and safety assessment framework for Iran’s military hospitals

Dimensions	Sub-dimensions	Indicators No.
**Clinical Effectiveness**	Conformity of processes of care	1–6
	Outcomes of processes of care	7–11
**Safety**	Patient Safety	12–19
	Health Worker Safety	20–25
**Efficiency**	Appropriateness of care	26–27
	Productivity (ratio of input to output)	28–31
	Use of capacities	32–35
	Financial performance	36–44
**Patient Centeredness**	Management and handling of patient complaints	45–46
	Patient satisfaction and experience management	47–47
**Responsive Management (Command and Control)**	Staff management	49–53
	Accidents and disasters Management	54–55
	Management and security of data and information	56
	Environmental safety management	57
	Management of combat medicine and military health	58

**Table 4:** Validation scores of the quality and safety assessment framework for Iran’s military hospitals

No.	Assessment Criteria	Agreement between participants (kappa coefficient)
1	Applicability of the framework	0.92
2	Adaptation of the developed framework to the upstream documents	0.92
3	Ability to accept the framework by stakeholders	0.92
4	Efficiency of the framework	1
5	Flexibility of the framework	1
6	Effectiveness of the framework	0.89
7	Simplicity of the framework	0.92
8	Coherence and integration between framework dimensions	1
9	Comprehensiveness of the framework	0.92
10	Overall	0.92

## Discussion

The Q&SAF for Iran’s military hospitals was developed through the utilization of a mixed-method approach and parallel use of review methods, quantitative, and qualitative methods. This framework has 58 quality and safety assessment indicators categorized under 15 sub-dimensions and 5 main dimensions, including clinical effectiveness, safety, efficiency, patient-centeredness, and Responsive Management (Command and Control).

Utilization of the indicators and dimensions identified from the literature and using the experiences of national and sub-national experts in developing the framework strengthened the study. Developing performance assessment frameworks using the qualitative studies approach and the Delphi technique and expert panel is a common and scientific way that has been used in many studies at different levels of the health system. Bruno et al. (2015) regarding the providing of guideline-based quality indicators for primary care in England, Veena et al. (2005) in the development of coronary artery bypass surgery quality indicators and also, Tabrizi et al. (2013) to develop performance indicators for patient and community engagement and to improve educational management in hospitals, have used the Delphi method and expert panel [36–39].

According to a review of several assessment frameworks that provided for hospital quality and safety, the primary challenges were related to the incompleteness of some frameworks and inability of some other to coverage all of hospital functional areas [40]. The Q&SAF for Iran’s military hospitals is sufficiently thorough and covers all functions, from clinical to administrative and financial. This important issue has been considered in the WHO-PATH framework and the American Medicare Hospital Comparison Program.

In accordance with most previous frameworks, the majority of the indicators utilized to assess the Iran’s military hospitals quality and safety were at the level of outcome assessment. The experts also believed that the results of the hospital’s performance should be quality-oriented and the framework should assess the results of the activities.

Based on the finding of literature review and comparative review of Q&SAF; The most focus on quality in hospital was the clinical effectiveness dimension, which is assessed in all of current frameworks [41]. This reflects the current trend toward adhering to clinical and evidence-based medical guidelines and highlights the significance of initiatives and methods for assessing the cost effectiveness of services [42]. Accordingly, clinical effectiveness has been considered in the Iran’s military hospitals Q&SAF, and 11 indicators have been assigned to it.

As frontline defenders, health workers are at high risk of infection during the COVID-19 pandemic [43–45]. The safety of health workers and patients is a unique advantage in the quality of healthcare and an important priority in healthcare systems [32, 41, 46]. The 13th general work plan of the WHO and the strategic vision of EMRO all prioritize the safety of health workers, and the WHO has considered September 17 as the World Patient Safety Day since 2019 [47, 48]. According to the reviewed frameworks (ACHS and QIP) which pay special attention to the safety dimension, in the Iran’s military hospitals Q&SAF, the patient and health worker safety were emphasized and 14 indicators have been assigned to safety dimension. Hospital efficiency is a lever to improve the development of a health care system. It is important for a hospital to maintain the level of quality in healthcare services while achieving efficient services at the lowest cost [49]. Military hospitals are financed annually through the Global budget [50]. The government’s budget deficit and financial challenges have increased pressure on Iran’s military hospitals to reduced costs [50]. Efficiency must be accurately monitored in order to identify improvements in healthcare productivity [51]. In order to improve the efficiency of military hospitals and in accordance with 9 frameworks extracted from the literature (out of 13 frameworks); efficiency dimension by the largest number of indicators was considered.

The mission of military hospitals is to enhance the health of military personnel by providing health support to a wide range of covered military personnel [50]. Military hospitals are tasked with caring for injured soldiers as well as offering routine medical care to active-duty military members, their families, and retirees [50]. Due to increasing the health literacy of patients and changing the needs of the population; the responsiveness of hospitals has faced fundamental changes. Therefore, the hospital’s response should be patient-centered and should consider the patient’s priorities, needs, values, and clinical decisions in providing health services [52]. Based on this and in accordance with the WHO suggestion regarding the centrality role of patients in the hospital and involving them in providing service processes; one of the important dimensions of the Iran’s military hospitals Q&SAF was assigned to the patient-centered dimension.

In addition to the many similarities that military hospitals have with civilian hospitals in providing health services to the community; in some functional aspects; due to the specific population coverage and specific missions, they have few differences with civilian hospitals [53]. Therefore, in the developed framework, it was necessary to define a specific dimension for military hospitals in accordance with its specific missions. Accordingly, the Responsive Management (Command and Control) dimension with the sub-dimensions of staff management, accidents and disasters management, management and security of data and information, environmental safety management and management of combat medicine and military health were considered. The assessment of these sub-dimensions will be done based on the specific guidelines that were used for military hospitals assessment. Using global experiences to assess the quality of hospitals and combining it with the specific missions of military hospitals can improve the performance of these hospitals similar to civilian hospitals.

The developed assessment framework and associated quality and safety improvement indicators can be tailored for use in civilian hospitals to enhance patient care. The applicability and adaptability of this framework in civilian hospitals can be greatly improved by considering key influencing factors, such as customizing the indicators to fit the local context to ensure their relevance and applicability, integrating them with existing information systems and reporting mechanisms, and conducting pilot tests to gather feedback and make necessary adjustments [54, 55].

The participation of patients and community could increase the comprehensiveness and effectiveness of the framework. One of the study’s limitations is the absence of patient engagement in the framework development process. In order to reduce the impact of this limitation, indicators related to the patient-centered dimension were included.

## Conclusion

The Iran’s military hospitals Q&SAF; as a comprehensive tool, provides a suitable opportunity for policy makers and managers to assess the hospitals quality and safety and formulate effective strategies to improve the hospital performance. It is suggested that this framework and its suggested indicators be used for the quantitative and qualitative assessment of Iran’s military hospitals, including the financial resources required to provide health services, human resource management, quality of care, patient and health worker safety, and other functional aspects. Also, this framework can be considered as a reference in assessing and comparing the performance of military hospitals.

## Data Availability

Data will be made available on request.
